# Ductal Carcinoma In Situ (DCIS) Diagnosed by MRI-Guided Biopsy among *BRCA1/BRCA2* Mutation Carriers

**DOI:** 10.1155/2022/4317693

**Published:** 2022-10-27

**Authors:** Renata Faermann, Eitan Friedman, Orit Kaidar-Person, Jonathan Weidenfeld, Malka Brodsky, Anat Shalmon, Osnat Halshtok Neiman, Michael Gotlieb, Yael Yagil, David Samoocha, Dana Madorsky Feldman, Miri Sklair-Levy

**Affiliations:** ^1^Meirav Center for Women's Health and High-Risk Clinic, Sheba Medical Center, Ramat Gan, Israel; ^2^Division of Diagnostic Imaging, Sheba Medical Center, Ramat Gan, Affiliated with the Sackler School of Medicine, Tel Aviv University, Tel Aviv, Israel; ^3^Sackler School of Medicine, Tel-Aviv University, Tel-Aviv, Israel; ^4^Department of Radiation Oncology, Sheba Medical Center, Ramat Gan, Israel; ^5^Department of Pathology, Sheba Medical Center, Ramat Gan, Israel

## Abstract

**Background:**

While *BRCA1/BRCA2* pathogenic sequence variants (PSVs) clearly confer an increased risk for invasive breast cancer, the extent to which these mutant alleles increase DCIS risk is less clear.

**Objective:**

To assess the rate of detection over a 5-year period, and MRI imaging features of pure noncalcified DCIS in a cohort of Israeli *BRCA1/BRCA2* PSV carriers attending a high-risk clinic from 2015 to 2020.

**Materials and Methods:**

All female *BRCA1/BRCA2* PSV-carriers followed at the Meirav High-risk clinic from 2015 to 2020 were eligible if they underwent semiannual breast imaging (MRI/mammography) and MRI-guided biopsy-proven pure DCIS. Clinical data, pathology information, and imaging characteristics were retrieved from the computerized archiving system.

**Results:**

18/121 (15.2%) participating *BRCA1* PSV carriers and 8/81 (10.1%) *BRCA2* PSV-carriers who underwent MRI-guided biopsy were diagnosed with DCIS. The median age of *BRCA1* carriers and *BRCA2* carriers was 49.8 years and 60.6 years, respectively (*p* = 0.55). Negative estrogen-receptor tumors were diagnosed in 13/18 (72%) *BRCA1* and 2/8 (25%) *BRCA2* PSV carriers (*p* < 0.05). Thirteen (13/18–72%) *BRCA1* carriers had intermediate to high-grade or high-grade DCIS compared with 4/8 (50%) of *BRCA2* carriers (*p* = 0.03). Over the 5-year study period, 29/1100 (2.6%) *BRCA1/BRCA2* PSV carriers were diagnosed with DCIS seen on MRI only.

**Conclusion:**

MRI-detected noncalcified DCIS is more frequent in *BRCA1* PSV carriers compared with *BRCA2* carriers, unlike the *BRCA2* predominance in mammography-detected calcified DCIS. *BRCA1*-related DCIS is diagnosed earlier, more likely to be estrogen receptor-negative and of higher grade compared with *BRCA2*-related DCIS. Future prospective studies should validate these results and assess the actual impact they might have on clinical management of *BRCA* PSV carriers.

## 1. Introduction

Women harboring germline *BRCA1* or *BRCA2* pathogenic sequence variants (PSVs) are at a substantially increased risk for developing breast (and ovarian) cancer (BC). While actual breast cancer risk differs by study design and case selection, the most comprehensive updated risk estimates are 72% and 69% lifetime risk for *BRCA1* and *BRCA2* PSV carriers, respectively [1].

While risk is clearly increased for invasive BC, the extent to which these mutant alleles are associated with an increased risk for DCIS is less clear. Originally, a lower incidence of pure DCIS or DCIS accompanying invasive BC in *BRCA1/BRCA2* PSV carriers was found compared with non-*BRCA* carriers/sporadic cases of BC [2–4]. In fact, DCIS was reportedly rare in *BRCA1* carriers, or even not considered as part of the cancer spectrum risk conferred by *BRCA1* PSV [2, 5]. In the Breast Cancer Linkage Consortium (BCLC), carriers of *BRCA1* mutations showed less DCIS around the invasive lesion compared with controls (sporadic BC cases) (41 vs 56%) [3]. More recent studies reported higher prevalence of DCIS incidentally diagnosed during risk reducing mastectomy in *BRCA1* and *BRCA2* PSV carriers—Kauff and coworkers compared *BRCA* carriers with age- and race-matched controls without a known cancer predisposition. They found 3 DCIS cases in 24 reducing mastectomy (13%) in *BRCA* carriers vs. none of the 48 controls—*p* = 0.034 [6]. Moreover, Bayraktar et al. [7] showed that 10% (12/118) and 17% (20/118) of their studied DCIS cases harbored *BRCA1* and *BRCA2* PSV, respectively. Hwang reported a cohort of 129 PSV carriers and 269 noncarriers. 48 (37%) PSV carriers had DCIS (with or without invasive cancer) compared with 92 noncarriers (34%). DCIS was as equally as prevalent in patients that are PSV carriers, to those that are noncarriers, but occurred at an earlier age in PSV carriers. [8]. The risk of developing invasive breast cancer after a diagnosis of DCIS ranges from 14% to 60% at 10 years [9]. When DCIS is diagnosed at an early age (premenopausal women), the prognosis is worse [9, 10].

In the Ashkenazi Jewish (AJ) population, a limited number of PSVs are reported in the *BRCA1* {c.5266dupC (p.Gln1756Profs) [5382insC], c.66_67AG (p.Glu23 fs)[185delAG]} and *BRCA2* {c.5946del (p.Ser1982 fs) [6174delT} genes. These 3 predominant PSVs have been reported to be detected in ∼2.5% in the general cancer-free AJ population [11] and 11-12% in unselected consecutive AJ BC cases [12, 13].

Pure DCIS may present as calcifications on mammography, diagnosed by vacuum-assisted biopsy. Mammography-detected calcifications diagnosed as pure DCIS are more commonly encountered in *BRCA2* PSV carriers than in *BRCA1* PSV carriers—Krammer et al. reported a cohort of 250 *BRCA1* and 246 *BRCA2* carriers. Pure DCIS was more encountered in *BRCA2* PSV carrier than *BRCA1* carriers. *BRCA2* mutation carriers presented with DCIS alone in 14% (35/246), whereas *BRCA1* mutation carriers presented with DCIS alone in 9% (23/250) (*p* = 0.0026) [14].

MRI is the most sensitive breast imaging tool, especially in young *BRCA1*/*BRCA2* PSV carriers, with a sensitivity between 75.2 and 100%, generally over 80%, and specificity between 83% and 98.4% [15]. MRI imaging of DCIS may reveal a host of imaging abnormalities: foci, non-mass enhancement, and masses.

MRI has reportedly outperformed either mammography or ultrasound in assessing the extent of disease in DCIS cases: MRI sensitivity for accurate assessment of DCIS extent was 89% compared with only 55% and 47% for mammography and ultrasound, respectively [16].

To the best of our knowledge, a comparison of the incidence of noncalcified DCIS between *BRCA1* and *BRCA2* mutation carriers, detected by MRI, has never been reported.

The aim of the current study was to assess the rate of detection over a 5-year period and the imaging features of MRI-detected pure noncalcified DCIS among Israeli *BRCA1/BRCA2* PSV carriers who attend a high-risk clinic in a single medical center between 2015 and 2020 and to compare the incidence of noncalcified DCIS by mutated gene.

## 2. Materials and Methods

All female *BRCA1* and/or *BRCA2* PSV carriers followed at the Meirav high-risk clinic, Sheba Medical Center, Tel HaShomer, Israel from 2015 to 2020 were eligible if they underwent regular breast imaging protocol offered (see below) and underwent an MRI-guided biopsy for a radiologically suspicious lesion. For each study participant, clinical data such as ethnicity, specific *BRCA* gene PSV, type and size of the imaging findings, grade of DCIS, and receptor status were retrieved from the computerized archiving system: Picture Archiving and Communication System (PACS), Radiology Information System (RIS), and the central database of the medical center (Camelon). For each MRI, a BI-RADS score for MRI result that required a biopsy (4 or 5) was assigned, and pathology reports were individually retrieved and reviewed.

Imaging protocol surveillance of *BRCA* PSV carriers in our institution during the study period included annual MRI starting at 25 years of age and adding annual mammogram starting at age 30 years, alternating with MRI so that breast imaging is performed every six months. Between the ages of 30 and 40 years, a one-view only mammography for each breast (MLO view) is performed to minimize ionizing radiation exposure. Between ages of 40 and 50 years, performing one- or two-view mammography depends on the radiologist's preference. Between 25 and 29 years of age, an ultrasound alternating with MRI is also performed. Any suspicious imaging finding on MRI, such as a non-mass enhancement (NME) or a focus, has led to a second-look mammography to look for microcalcifications. If a mass or a large NME was detected, a second-look ultrasound was also performed. MRI-guided vacuum biopsy was performed when there was no mammography or ultrasound correlate with the MRI finding.

Breast MRI scans were performed on a 1.5-T system (Signa Excite HDx; GE Healthcare) with a dedicated double breast coil equipped with 8 channels. A standard dynamic contrastenhanced MRI protocol was via axial vibrant multiphase 3D DCE T1-weighted sequence with the following parameters: echo time (TE)/ repetition time (TR) of 2.6/5.4 ms; flip angle 15°; bandwidth 83.3 kHz; matrix, 512 × 364; field of view (FOV) 340 mm; slice thickness of 2 mm. Images were acquired prior to and four times after an automated injection of contrast agent bolus (0.1 ml/kg at 2 ml/sec Dotarem (gadoterate meglumine, Guerbet)) followed by a 20-ml saline flush. Postcontrast images were acquired, with the first acquisition centered at 1 : 25 minutes after injection and the delayed acquisition centered at 7 : 35 minutes after injection, as was previously described [17].

All MRI-guided biopsies were performed using a Sentinelle Vanguard™ Breast MRI coil in GE Signa 1.5 T scanner (described above), with a vacuum-assisted biopsy needle (Hologic Suros ATEC® Sapphire breast biopsy system for stereotactic biopsy).

All MRI-guided biopsies performed during the study period in our institution in the context of the high-risk clinic were retrieved and reviewed by two independent physicians. Those with a pathology result of pure DCIS (meaning only ductal carcinoma in situ, without the evidence of invasive component) were included in the study. Patients with calcifications in a mammography before the MRI biopsy were excluded from the study.

All MRI and mammography interpretation and MRI-guided biopsies were carried out by a fellowship-trained radiologist.

### 2.1. Statistical Analysis

Comparison of the rate of DCIS between groups, comparison of negative estrogen receptor, and number of intermediate to high-grade and high-grade DCIS, as well as the median size of the lesions, were performed using Fisher's exact test. All statistical analyses were calculated using SPSS V27. *P*-values were considered significant at *p* ≤ 0.05.

This study was ethically approved by the Sheba Institutional Review Board with an exemption from obtaining written informed consent from the study participants.

## 3. Results

Overall, during the study period (2015 to 2020), 1100 *BRCA* PSV carriers were eligible for participation: 629 *BRCA1* PSV carriers and 447 *BRCA2* PSV carriers, and in the remaining cases (*n* = 24) the mutated gene could not be clearly ascertained, or they carried a PSV in both genes. During the study period, 227 MRI-guided biopsies were performed in 209 *BRCA* PSV carriers: 121 *BRCA1* carriers, 81 *BRCA2* carriers, 2 were double *BRCA1* and *BRCA2* PSV carriers, and in 5 the mutated gene could not be ascertained.

**Figure 1:**
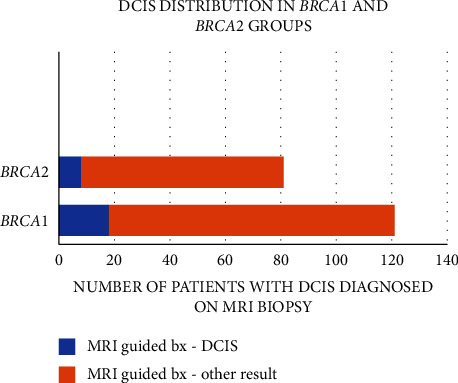
BRCA patients that underwent MRI-guided biopsy; part of them had the diagnosis of DCIS on MRI biopsy divided by groups.

During the study period, 29/209 biopsied different cases were diagnosed as DCIS, all not calcified on mammography. In two of these cases, patients harbored both *BRCA1* and *BRCA2* PSVs and were excluded from further analysis, and an additional case (with a nonassignable mutated gene) was lost to follow-up. Thus, subsequent analysis was focused on the remaining 26 DCIS cases. Of 121 *BRCA1* PSV carriers who underwent biopsy, 18 were diagnosed with DCIS (15.2%), and 8/81 *BRCA2* PSV carriers were also diagnosed with DCIS (10.1%) (*p* > 0.05) (Figure 1).

Over the 5-year study period, 29/1100 (2.6%) *BRCA1/BRCA2* PSV carriers were diagnosed with DCIS seen on MRI only.

Relevant clinical and tumor specific features (e.g., median age at diagnosis, estrogen receptor status, and grade of DCIS) by mutated gene are shown in Table 1.

Thirteen (13/18—72%) *BRCA1* PSV carriers and 2/8 (25%) *BRCA2* PSV carriers had negative estrogen receptor (ER) (*p* < 0.05). Among *BRCA1* carriers with ER-negative, one of them was progesterone receptor (PR)-positive, and the rest were ER- and PR-negative.

**Figure 2:**
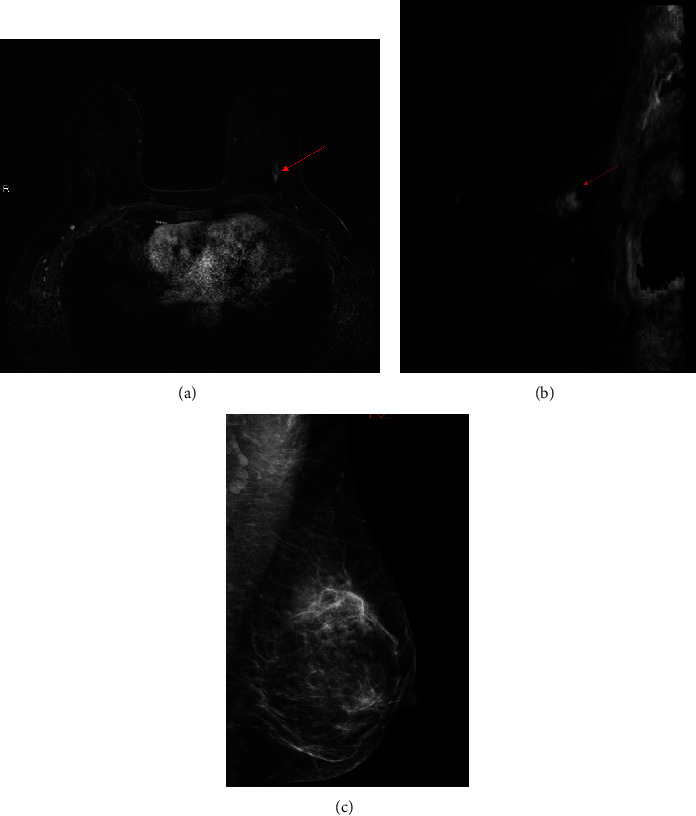
40 year-old BRCA1 patient undergoing annual MRI. Axial (a) and Sagittal (b) reconstruction showing a segmental nonmass enhancement in the left breast (red arrow). Mediolateral (MLO) view of mammography of the same breast shows no corresponding calcifications.

**Figure 3:**
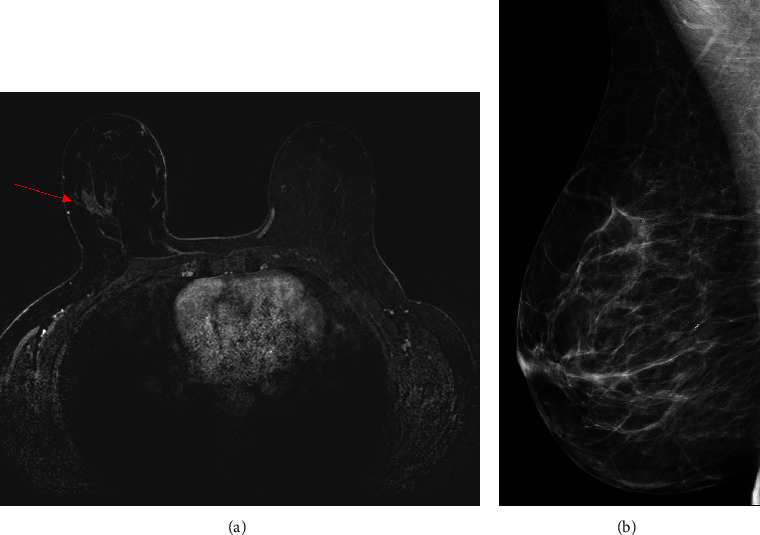
43 year-old BRCA2 carrier, undergoing annual MRI, showing a new NME in the right breast (A axial image, red arrow). MLO view of the right breast shows no corresponding calcifications.

MRI imaging findings were mainly non-mass enhancement (NME)—10/18 (55%) in *BRCA1* PSV carriers and in all (8/8-100%) *BRCA2* PSV carriers. These findings are specified in Table 2, and examples are shown in Figures 2 and 3.

Fifteen (15/18—83%) of *BRCA1* PSV carriers diagnosed with DCIS had high grade or intermediate to high-grade DCIS, while 4/8 (50%) of the *BRCA2* PSV carriers had these DCIS grades.

The size range of the MRI finding in DCIS-positive cases was 3 to 70 mm in *BRCA1* (median size 14.5 millimeters) and 8 to 100 mm in *BRCA2* (median size 14 mm) (*p* > 0.05).

## 4. Discussion

To the best of our knowledge, the current study is the first to report the rates of MRI-only diagnosed occult DCIS in *BRCA1/BRCA2* PSV carriers—2.6% (29/1100) over a 5-year surveillance in a single medical center in Israel, and the rate of biopsy-diagnosed “pure” DCIS was 13.9% (29/209).

Previous studies on pure DCIS or DCIS adjacent to IDC diagnosed by any breast imaging technique (primarily mammography) reported that these diagnoses were more prevalent in *BRCA2* PSV carriers than in *BRCA1* PSV carriers—Arun et al. analyzed 73 *BRCA1/2* PSV carriers—70% of *BRCA2* carriers had preinvasive lesion adjacent to the IDC, in comparison to 52% of *BRCA1* [18].

Rijnsburger et al. reported the MRI sensitivity was superior to that of mammography for invasive cancer (77.4% *v*35.5%;*P* < 0.00005) but not for DCIS [19]. In contrast, for DCIS cancers only, the sensitivity of mammography (69.2%) was much higher than that of MRI sensitivity (38.5%) in their study. The present study, that is based on MRI imaging only, shows that noncalcified DCIS seen on MRI is not rare, implying another important role of MRI imaging on PSV mutation carriers, in contrast to Rijnsburger's study.

Warner et al. [20] reported that all 6 out of 236 *BRCA* carriers diagnosed with pure DCIS were *BRCA2* PSV carriers, of which 3 were diagnosed only by MRI. Similarly, the study by Liu and co-workers [21] reported that among women diagnosed with DCIS and genotyped for *BRCA* PSVs, the incidence of DCIS in *BRCA2* PSVs was higher than that of *BRCA1* PSV mutation (27.5%—8/29 versus 3%—1/33) (*p* = 0.009) [21]. These data are inconsistent with the data presented herein, where most DCIS cases were diagnosed in *BRCA1* PSV carriers. The inconsistent results may stem from the lack of calcifications of the tumors reported herein and targeting MRI-only detected tumors compared with previous studies that have analyzed all DCIS-diagnosed cases, including those that were detected by mammography and those that were IDC adjacent. Indeed, *BRCA2*-associated DCIS are more likely to present with calcifications that are detected by mammography [14]. In the present study, calcified tumors were excluded from the analysis. Thus, the current study establishes the fact that the absence of calcifications on mammography does not indicate that *BRCA1* PSV carriers do not develop DCIS, only that its radiological features may be different from those in *BRCA2* carriers.

Yang et al. reported that pure DCIS was diagnosed in 15/71 *BRCA1* patients (21.1%) undergoing surgery for BC and 10/43 *BRCA2* patients (23.3%), in addition to DCIS associated with IDC diagnosed in both carrier groups (45/71—63.4% and 26/43—60.5%, respectively) [22]. Thus, most BRCA-associated invasive BC had adjacent DCIS, arguing for a DCIS-associated premalignant pathway. Originally, DCIS-associated preinvasive pathway was presumed to be absent in *BRCA*-associated BC, in contrast to sporadic BC [3]. However, later studies showed pure DCIS and IDC associated with DCIS in *BRCA*-associated BC, supporting the notion that DCIS is a precursor to invasive BC in *BRCA* PSV carriers, like sporadic cases [22]. Furthermore, it has also been initially reported that DCIS is less often found with IDC in *BRCA* PSV carriers compared with sporadic cases [2–4]. Yet, subsequent studies showed that DCIS can also be found in risk-reducing mastectomy in *BRCA* PSV carriers [6, 23–25]. Currently, and consistent with the study reported herein, it is well established that *BRCA* PSV carriers may be diagnosed with DCIS-only cancers.

Our study shows that *BRCA1* PSV carriers also develop DCIS that can be detected by MRI and not only by mammography. The present MRI-only based study, that demonstrated that noncalcified DCIS is not rare in *BRCA* PSV carriers, further supports the importance of periodic MRI imaging as an effective screening modality in *BRCA* PSV carriers. These MRI effectiveness results in detecting DCIS in *BRCA* PSV carriers are in line with a previous study by Warner [26], who reported that 10/445 *BRCA* PSV carriers were diagnosed with DCIS by MRI (4/10—*BRCA1* PSV carriers and 6/10—*BRCA2* PSV carriers).

Importantly, phenotypic and clinical differences were noted by the mutated gene: DCIS was diagnosed at a younger age (not statistically significant), with a higher rate of intermediate-to-high-grade DCIS and ER negativity in *BRCA1* PSV carriers compared with *BRCA2* PSV carriers. These features and differences in phenotypes between female carriers of the two mutated genes have also been reported for invasive BC [19], further supporting the notion that DCIS can be regarded as a precursor for invasive BC.

In the current study, MRI-detected DCIS was diagnosed at a younger age and higher grade in *BRCA1* carriers than in *BRCA2* carriers. Krammer et al. reported similar mean age of both invasive and DCIS diagnoses in *BRCA1* (44.1 years) and *BRCA2* carriers (45.1 years). Hwang reported a median age of 59 years in *BRCA1* and 49 years in *BRCA2*, for any type of breast cancer. Since *BRCA1* carriers usually have a higher-grade disease, and more frequently hormone-negative, diagnosing DCIS earlier would be extremely important to try to prevent these carcinomas evolving into an invasive disease.

Krammer et al. showed that breast cancers in *BRCA1* mutation carriers are associated with more aggressive tumor characteristics than that of *BRCA2*, similar to our study, and are less well seen on mammography [14]. In patients that undergo annual MRI, the objective of mammography is to detect calcifications that could represent DCIS that was not seen on MRI. Since mammography rarely identified cancers not visible on MRI in their study, and since our study shows MRI-detected DCIS seen more commonly in *BRCA1* than *BRCA2* patients, the omission of mammography in *BRCA1* mutation carriers screened with MRI that was suggested in their study might be extrapolated to our study, although this comparison is limited since we did not include DCIS diagnosed with mammography. *BRCA1* carriers are sensitive for radiation; therefore, we suggest that mammography could be omitted, or it could be performed once every two years, with a one view only.

The current study has obvious limitations and caveats. It is a retrospective, single-center study, with a small number of patients diagnosed with DCIS and a short follow-up. Yet, the fact that the spectrum of PSV is limited in Israeli (mostly Jewish) *BRCA* PSVs and the fact that the surveillance scheme was carried out by the same team using the same equipment help in reducing any biases but does not eliminate them in total.

In conclusion, noncalcified MRI-only diagnosed DCIS is an important entity that is diagnosed by adhering to an annual MRI screening of female *BRCA* PSV carriers, more frequently in *BRCA1* than in *BRCA2*, and in these *BRCA1* PSV carriers, the tumor is more likely to be estrogen receptor negative and displays high-grade phenotype. If these data are validated in an extended study, these realities that may clinically translate to a more aggressive treatment approaches to these tumors should be discussed with female *BRCA* PSV carriers during their follow-up sessions.

## Figures and Tables

**Table 1:** Age distribution, DCIS grade, and hormone receptors.

	BRCA 1 [18]	BRCA 2 [8]	*p*
Median age	49.8 ( ± 13)	60.6 ( ± 11)	0.055
Negative estrogen receptor	13 (72%)	2 (25%)	<0.05
High-grade/intermediate to high-grade DCIS	13 (72%)	4 (50%)	0.03

**Table 2:** MRI imaging findings.

	*BRCA 1* (number of patients)	*BRCA 2* (number of patients)
Linear nonmass enhancement	1	5
Segmental nonmass enhancement	2	—
Nonspecified NME	6	2
Focus	5	1
Mass	4	—

## Data Availability

Access to data is restricted due to patient privacy, as per the regulations of the ethics committee that approved this study.
